# Supplementary material for: Are carbon emissions trading and green financial instruments synergistic? -Comprehensive quantitative research based on content analysis
Source: PLoS One. 2024 Mar 7;19(3):e0298601. doi: 10.1371/journal.pone.0298601 (PMC10919601; doi:10.1371/journal.pone.0298601)
Supplement: S1 Data — (ZIP) [file pone.0298601.s001.zip › Data/地级市Z_F工作报告.zip!/║╙─╧/┐¬╖Γ╩╨/└┤╫╘╣1⁄2╓┌║┼í╛┬φ┐╦╩2╛▌═°í┐_2017.docx]

各位代表：

　　现在，我代表市人民政府，向大会报告工作，请予审议，并请各位政协委员和列席人士提出意见。

　　一、2016年工作回顾

　　过去的一年，面对复杂严峻的经济形势，在省委、省政府和市委的坚强领导下，在市人大、市政协的监督支持下，全市上下深入学习贯彻习近平总书记系列重要讲话精神，认真落实市第十一次党代会部署，以新发展理念为引领，坚持“1234”总体思路，坚持“稳中求快”总基调，持续抓培育、扩增量、上项目、强支撑、惠民生，全市经济社会实现平稳健康较快发展，实现了“十三五”良好开局。

　　（一）稳增长保态势，经济社会实现平稳健康较快发展。从发展速度看，全市生产总值完成1748亿元，增长8.5%，增幅居全省第5位；社会消费品零售总额841亿元，增长12.6%，增幅居全省第2位；一般公共预算收入113.2亿元，增长10.2%，增幅居全省第5位；规模以上工业增加值625亿元，增长9%，增幅居全省第5位；固定资产投资1526.6亿元，增长15.3%，高于全省平均水平1.6个百分点，主要经济指标增幅均高于全省平均水平。从发展质量看，三次产业结构16.4∶40.2∶43.4，三产比重提高2.2个百分点。税收占一般公共预算收入比重达69%。高成长性、高新技术企业增加值分别增长11.3%和13.9%。人民币各项存贷款余额分别较年初增加188.9亿元、170.5亿元，新增存贷比90.3%。新业态、新模式发展迅速，新登记市场主体增长16%，电子商务交易额增长30%以上。房地产开发投资完成212亿元，增长25.9%。民间投资1343.2亿元，增长15.9%，增幅居全省第2位。非公有制经济增加值1109亿元，对经济增长贡献率达到55%，拉动经济增长4.7个百分点。从经济效益和社会效果看，单位生产总值能耗下降4.8%。常住人口城镇化率45.5%，提高1.3个百分点。城乡居民人均可支配收入达到24596元和11166元，增长7.3%和8.4%，增幅分别居全省第2和第3位。居民消费价格指数增长1.6%，保持基本稳定。事故发生起数、伤亡人数分别下降51.7%、60.8%。赴京、到省集访批次分别下降42.2%、25.5%。

　　（二）推进供给侧结构性改革，加快产业转型升级。制定了制造业、现代服务业、种养业供给侧结构性改革行动方案，研究出台补齐产业短板系列工作方案。持续稳定工业增长。成立市企业投诉中心，开展“政策落实进千企”活动，为企业减税降费8亿元；组织银企对接会6次，对接资金58亿元；发放还贷周转金203笔、22.4亿元；新增新三板挂牌上市企业9家。全市规模以上工业企业达到1091家，30个工业行业25个实现稳定增长；工业投资完成853亿元，增长20.6%，增幅居全省第4位，中联重科智能大马力拖拉机、奇瑞汽车K60新车型等一批工业新产品相继上市，电镀金刚线、石墨负极材料等一批重大工业项目建成投产。不断巩固农业基础。新建高标准粮田35万亩，新增改善有效灌溉面积30万亩，粮食生产再获丰收，总产达283.9万吨。现代农业产业化集群增加到19个，省级农业产业化龙头企业达到21家。新认定4家农业特色产业科技示范基地，新认定无公害农产品生产基地24个、无公害农产品34个。新增国家级标准化养殖场5家、省级生态养殖示范场8家，畜牧业综合评定居全省第2位。家庭农场达到1058家。农业机械化综合水平达到80.6%。提升服务业发展水平。积极培育消费新业态，建成中原明珠文化产业园、半亩园、万膳街、朱仙镇国家文化生态旅游示范区一期等一批文商旅项目，完成马道街、二马道街综合整治，双龙巷历史街区改造、文化交流中心等项目有序推进。恒大、万达、绿城、碧桂园等一批知名企业入驻开封，一批城市综合体相继开工建设。成功举办“两节一赛一论坛”、第十四届国际茶文化研讨会等重大活动。文化旅游产业持续升温，全年接待游客5080万人次，增长13.1%，旅游综合收入398.6亿元，增长62.6%。商贸、物流、邮政、电信、交通运输业健康发展。文化产业增加值占GDP比重达到5.6%，居全省第2位；第三产业增加值增长10.8%，增幅居全省第4位。

　　（三）形成合力破难题，打好“三大攻坚战”。强力推进脱贫攻坚。坚持精准扶贫、精准脱贫，有效落实金融、交通、水利、教育、卫生、电力等行业脱贫政策，统筹各类资金3亿元，落实 “十不十有” 精准脱贫标准，52个贫困村、2.46万建档立卡贫困人口达到脱贫标准，超省下目标7.5%。打好大气污染防治攻坚战。强力推进“六控”，实施烟花爆竹禁放，完成“国五”油品提升，全面取消禁燃区内所有散煤销售点，拆改建成区10蒸吨/时及以下燃煤锅炉158台，淘汰黄标车、老旧车31572辆，PM10浓度均值122微克/立方米，PM2.5浓度均值72微克/立方米，优良天数227天，居全省第2位。稳妥推进国企改革。研究出台《进一步深化国有企业改革的实施意见》，国有企业三项制度改革、国有企业负责人薪酬制度改革、投融资企业改革、国有影剧院等经营性文化事业单位转企改制扎实推进。新组建开封粮食产业集团、水务开发建设公司、智慧城市发展有限公司。驻汴央企、省企“三供一业”分离移交工作进展顺利。

　　（四）蓄势新动能，创新驱动和战略平台建设步伐加快。注重对事关全市发展全局性、根本性、长远性重大战略问题的谋划。加快战略性规划和平台实施。中国（河南）自由贸易试验区开封片区成功获批，开封从内陆城市一跃跨入改革开放最前沿。开港经济带、郑汴产学研结合示范带、沿黄生态带纳入省“十三五”规划盘子，“四带三区”产业布局初步展开，郑汴港“金三角”共识初步形成，郑汴一体化深度融合迈上新台阶。成功申建开封国家产城融合示范区。开封市被纳入中国（郑州）跨境电子商务拓展城市。加快创新驱动。全市科技支出3.1亿元，增长26%。成功申建2个省级高新区，实现省级高新区“零”的突破。培育众创空间、星创天地、科技企业孵化器18个。建立大学科技园，建成国家成套空分设备质量监督检验中心。实施“四个一”人才工程，深化与天津大学、中科院过程研究所、“千人计划”专家联谊会等高校及科研院所合作，挂牌成立“千人计划”工作站、启源先进光源产业研究院、开封文化产业研究院、天开研究院等创新创业平台，建成新型研发机构6家，新建院士工作站2个、市级以上工程技术研究中心23家，引进一批带技术、带项目、带资金高层次人才。新认定高新技术企业16家，科技型中小微企业达到403家。发展后劲持续增强。实施重大项目推进“六项机制”，“321”工程406个重点项目累计完成投资1012亿元，是近年来项目投资规模最大的一年，建成优德医疗设备产业园、中意湖、智慧城市展示体验中心等一批重点项目。创造PPP “开封模式” ，开港大道、涧水河等9个PPP项目进入执行阶段，其中5个项目入选财政部全国示范项目。夯实发展载体平台。服务业“两区”业态进一步完善，鼓楼区、尉氏县特色商业区分别荣获“十先”和“十快”称号。7个产业集聚区固定资产投资、规模以上工业增加值分别增长24.9%和11.6%，增幅分别居全省第5和第7位；新开工亿元以上项目288个，增加71个，汴西和杞县产业集聚区分别荣获全省“十强”和“十快”称号。

　　（五）深化改革扩大开放，激活发展内生动力。持续深化改革。深化行政审批、商事制度等“放管服”改革，公布权责清单，全面实施“五证合一”，新建30个“市民之窗”便民自助服务平台。基本完成农村土地承包经营权、农村宅基地和集体建设用地使用权确权登记颁证，启动农村产权制度改革试点。户籍制度改革和居住证制度实现“双落地”。完成公务用车制度和交通执法、食品药品监管等体制改革，启动不动产统一登记制度、出租车行业等改革。全面实施县以下机关公务员职务与职级并行制度。财税、投融资、金融、就业、教育、医疗卫生、科技等领域改革有序推进。持续扩大开放。扎实开展重点产业集中招商活动，成立汴商联合会，成功举办首届汴商大会，新引进省外项目242个，实际到位省外资金476.7亿元，增长8.2%，增幅居全省第7位；重大招商引资“532”工程引进项目35个，开工34个。进出口完成4.1亿美元，实际利用外资增长5.8%，增幅居全省第3位。

　　（六）推进新型城镇化，城乡面貌持续提升。深入落实中央城市工作会议精神，统筹抓好城市规划建设管理工作。加强规划设计。完成开港经济带概念规划、海绵城市及地下综合管廊规划、“一渠六河”景观设计等专项规划，11个片区控制性详规实现全覆盖，“两核六组团”空间布局规划更加完善。加快八网基础设施建设。郑徐高铁通车运营，郑民高速二期开封段建成通车，连霍高速开封站、郑民高速南苑站出入口完成拓宽改造，开尉路、开杞路完成主路段改造提升，郑开城际铁路延长线、机西高速二期等一批重大交通项目开工建设。开工夷山大街（土城段）、迎宾路（省府西街—西门大街）等“断头路”建设，完成内顺城路（龙亭北路—万岁山南口）、劳动路北延、孝严寺街、法院街等道路主路段改造。新增新能源纯电动公交车610台。加强黑臭水体治理，出境河流断面综合达标率88.9%；完成黑池、柳池饮用水源地清淤年度任务，饮用水源水质达标率100%。新建燃气管线77公里。新增供热1.8万户、390万平方米。新增35千伏及以下供电线路1135千米，全市供电能力达到606万千瓦。新增造林面积9万亩，高标准完成四条高速及开港大道等主要道路绿化。提升城市法治化和精细化管理水平。积极宣传、认真执行《城市市容和环境卫生管理条例》，开展“16个城市乱象” 集中整治，实施“拆违”行动，拆除违法建筑20.4万平方米。巩固国家卫生城市创建成果，积极创建全国文明城市，成功创建国家旅游标准化示范城市、国家园林城市，再添城市新名片。城乡面貌进一步改善。城乡一体化示范区完成基础设施投资37.5亿元，东部新城改造提升12条道路，各县县城面貌持续改观。继续开展“清洁家园·美丽乡村”活动，创建美丽宜居乡镇30个、美丽宜居村庄100个；改造农村危房6814户；克服困难，顺利完成农村公路三年行动计划，新改建农村公路1932公里，改造危桥3373延米，农村生产生活条件持续改善。

　　（七）办好民生实事，持续保障和改善民生。累计投入民生资金215.5亿元，占一般公共预算支出的72.8%，增长12.9%，十项民生工程50件为民实事全部完成44件，基本完成3件，3件推迟至今年实施。就业形势总体稳定。新增就业6.83万人，转移就业3.67万人，登记失业率控制在3%以内。提升社会保障水平。各项社会保障待遇按时足额发放，覆盖全民的社会保障体系基本建立。城乡低保分别提高至400元/月和2960元/年，农村五保集中供养和分散供养年人均标准分别提高至4000元和3000元。新增养老机构5家，床位1640张。24个棚户区、城中村改造项目扎实推进，保障性安居工程完成7592套（户）。提高教育水平。实施学前教育（二期）三年行动计划，新建、改扩建幼儿园44所；扩充城镇教育资源，新建中小学校5所、改扩建11所；关爱留守儿童，新建寄宿制小学31所；完成投资4.5亿元，改善农村义务教育薄弱学校办学条件；职教园区入驻7所院校3.5万名师生。各学段教育质量和内涵不断提升，教育改革扎实推进。顺河回族区通过义务教育发展基本均衡县（区）创建国家评估认定。提升医疗卫生健康服务能力。新建、改扩建医疗卫生机构25家，新增开放床位1933张，新建基层中医馆29个。扎实开展13项基本公共卫生服务，基本公共卫生服务经费标准提高至人均45元。初步建立分级诊疗制度。实施“一病两方”制度，开展医养结合工作。新农合参合率99.5%，人均年筹资水平达到540元。实行基本药物制度，采购配送率92%。落实全面“二孩政策”。中心医院新院区、妇儿医院、妇幼保健院病房楼等项目前期进展顺利。丰富群众精神文化活动。开展“政府购买公益演出”等系列文化惠民活动，全市公共图书馆、文化馆、乡镇综合文化站均免费开放。市体育中心完成招标，建成城墙体育公园及240处农民体育健身工程，全民健身活动蓬勃开展。有效防范化解风险。严格落实安全生产责任制，深化重点行业领域治理整顿，安全生产形势总体稳定。加强食品药品监管，食品药品安全形势稳定向好。做好非法集资稳控工作，金融秩序总体稳定。出台《进一步加强反恐怖工作的实施意见》，强化社会治安防控体系建设，严厉打击违法犯罪，人民群众安全感持续提升。普法宣传、人民调解、行政调解、法律援助工作顺利推进，社会大局保持和谐稳定。

　　一年来，我们坚持不懈加强政府自身建设，认真开展“两学一做”学习教育和“一学两争三优”活动，持续推进反腐倡廉建设，涌现出王振立等一批先进典型。坚持依法行政，自觉接受人大法律监督、政协民主监督，认真办理人大代表建议和政协提案，支持民主党派、工商联和无党派人士参政议政，接受社会各界工作监督。持续加强国防教育、国防后备力量及人民防空建设，驻汴解放军、武警部队和民兵预备役人员为开封发展做出了重要贡献，军民融合深度发展，第七次荣获“全国双拥模范城市”称号。民族文化博物馆成功创建全国民族团结进步教育基地。妇女儿童、残疾人、红十字、慈善等事业健康发展。宗教、统计、外事、侨务、对台、史志、档案、气象、地震、社科研究、对口援疆等工作都取得了新成绩。

　　各位代表！

　　过去一年取得的成绩来之不易。这是在经济形势异常严峻复杂、外部竞争异常激烈的条件下取得的，是省委、省政府和市委正确领导、科学决策的结果，是市人大、市政协有效监督、大力支持的结果，是全市人民负重前行、滚石上山的结果。在此，我代表市人民政府，向工作在全市各条战线上的广大工人、农民、知识分子、干部，向驻汴解放军指战员、武警官兵和公安民警，向各民主党派、工商联及社会各界人士致以崇高敬意！向所有关心、支持开封发展的同志们、朋友们表示诚挚的感谢！

　　在肯定成绩的同时，我们也清醒地看到，我市经济社会发展中还存在不少矛盾和问题：一是经济实力不强。总量小、底子薄、基础弱、人均水平低的基本市情尚未根本改变，生产总值、一般公共预算收入总量晋位升级面临很大压力。二是三次产业结构不优。一产比重偏大、二产比重偏低，骨干龙头企业少，产业层次不高；三产拉动作用虽逐年提升，但现代服务业比重偏小，调结构任务依然繁重。三是新旧动能转换动力不足。科技创新投入、创新人才引进、创新平台建设、创新型企业培育步伐不快，新业态新模式新动能培育尚需时日。四是风险隐患聚集显现。部分企业负债率高、亏损严重，潜在经营风险凸显；不良贷款增加，潜在信用风险上浮；社会稳定潜在风险有所抬头。五是现代化治理能力不强。政府职能转变不到位，改革任务艰巨；依法行政能力有待提高，公务员能力素质不能完全适应新形势、新要求。对此，我们一定勇于担当、切实解决。

　　二、2017年工作总体要求和目标任务

　　2017年，是党的十九大召开之年，扎实做好今年政府各项工作，意义深远、责任重大、使命光荣。今年政府工作的总体要求是：全面贯彻党的十八大和十八届三中、四中、五中、六中全会精神，更加紧密地团结在以习近平同志为核心的党中央周围，深入贯彻习近平总书记系列重要讲话精神，认真落实中央、省委经济工作会议和市第十一次党代会各项部署，坚持“1234”总体工作思路，坚持“稳中求快”总基调，突出以新发展理念为引领，突出以推进供给侧结构性改革为主线，突出以产业补短板为重点，突出以提高发展质量和效益为中心，着力稳增长、促改革、调结构、惠民生、防风险，促进经济平稳健康发展和社会和谐稳定，在决胜全面小康、加快建设“四个开封”的新征程中迈出坚实步伐，以优异成绩迎接党的十九大胜利召开。

　　主要预期目标是：生产总值增长8.5%以上，一般公共预算收入增长10%左右，规模以上工业增加值增长9%，固定资产投资增长15%左右，社会消费品零售总额增长12%左右，进出口总值增长5%，实际到位省外资金增长7%，实际利用外资增长5%，城镇新增就业5.5万人以上，常住人口城镇化率提高1.5个百分点左右，居民消费价格指数涨幅3%以下，节能减排、环境保护等约束性指标完成省下达任务。保持“三个同步”，即城乡居民收入与生产总值同步增长，生态环境质量与经济质量效益同步改善，社会事业进步与经济发展水平同步提高；实现“三个高于”，即生产总值、财政收入、居民收入增速均高于全国、全省平均水平，使决胜全面小康、建设“四个开封”的步伐更加稳健、更加坚实。

　　落实总体要求，政府工作在大局把握上，要妥善处理好“十个关系”。一是处理好“稳”与“快”的关系，在“稳”的基础上实现经济较“快”增长；二是处理好增长速度与质量效益的关系，既保持较快的增长速度，更要提升发展的质量和效益；三是处理好实体经济与虚拟经济的关系，做到既抓实体经济不放松，又把虚拟经济做活，两者齐头并进；四是处理好传统产业与新兴产业的关系，抓住传统产业、做强存量，抓好新兴产业、做大增量；五是处理好扬长与补短的关系，把优势产业做得更优，逐步补齐产业发展短板；六是处理好适度债务与加快融资的关系，把加快融资放在首要位置，同时兼顾债务风险防控；七是处理好粮食作物与经济作物的关系，在保证粮食安全的前提下，发展优质经济作物；八是处理好贫困村与非贫困村贫困户脱贫的关系，在抓好贫困村脱贫的基础上，着力抓好非贫困村贫困户脱贫工作；九是处理好抓文化与抓工业的关系，坚持文化和工业“双轮”驱动，坚定新型工业化城市和国际文化旅游名城两个定位；十是处理好老城改造和新区发展的关系，既抓好老城区改造，也抓好新区建设，形成“古今文明交相辉映，老城新区各展风采”的开封特色。

　　完成预期目标，政府工作在方法和路径上，要坚持“五个不变”、实现“五个转变”、聚焦“五个重点”。坚持“五个不变”：一是坚持推进中央及省决策部署在我市的落实不变；二是坚持“稳中求快”的总基调不变；三是坚持“双轮”驱动、“双+”互动、“双城”融合的战略举措不变；四是坚持“四带三区”、“两核六组团”的布局不变；五是坚持抓培育、扩增量、上项目、强支撑、惠民生的着力点不变。实现“五个转变”：一是在项目推进上实现从全面谋划向重点突破转变，务必扩大有效投资；二是在产业培育上实现从产业聚集向产业素质、产业融合转变，务必加快产业转型升级；三是在城市建设上实现从重中心城区向城乡统筹区域协调转变，务必加快新型城镇化步伐；四是在动力支撑上实现从传统拉动向创新驱动转变，务必增强发展内生动力；五是在工作推进上实现从部门“单兵突击”向“协同攻坚”转变，务必打好“四大攻坚战”。聚焦“五个重点”：一是聚焦“装备制造业及新兴产业、生产性服务业、现代农业、四种经济新业态”等重点产业抓培育，力争在补短板上有突破；二是聚焦“自贸区开封片区、郑汴深度融合、开港经济带”等重点片区扩增量，拓展发展新空间；三是聚焦“两改两拆、双提双修”等重点环节促协调，全面提升城乡规划建设管理水平；四是聚焦“创新驱动、改革推动、项目拉动、开放带动、风险防控”等关键领域强支撑，增强发展动力与活力；五是聚焦“脱贫、就业、环境、入学、就医、出行”等热点难点问题惠民生，让人民群众共享“四个开封”建设新成果。

　　三、全面做好2017年工作

　　把握总要求、实现预期目标，重点抓好以下五个方面的工作。

　　（一）补短板、求突破，加快产业转型升级。以供给侧结构性改革为主线，深入落实补齐产业短板各专项方案，打好发展转型攻坚战，推动产业向中高端发展。

　　推进工业经济提质增效。传统产业改造升级。化工产业要向精细化发展，依托晋开、东大、久龙等龙头企业，壮大煤化工、精细化工产业链，抓好精细化工产业集聚区建设，启动晋开公司一期搬迁，做好三期建设前期工作，加快东大化工搬迁。纺织服饰产业要完善体系，依托福甬服装、洛赐菲纺织机械等龙头企业，做强棉纺、做大服装、做优印染、补缺织造，推动形成纺、织、染、服装等完整产业体系。食品工业要提升综合实力，依托福润、海壹、禾丰、汇源、大用等龙头企业，做强面制品、肉制品、速冻食品，提升油料加工、果蔬加工、休闲食品、特色食品竞争力，提升大蒜、花生深加工产品的品质、品相、品牌，叫响金杞大蒜、开封花生等农产品品牌。主导产业重点突破。装备制造及汽车和零部件产业要提升智能化水平，推进空分产业向深冷、空气净化、环保配套等领域转型，促进汴西汽车零部件产业园与海马、日产、宇通等企业合作，支持中联重科大马力智能拖拉机项目扩大市场，力争奇瑞等新能源汽车入驻开封。加快优德医疗设备产业园建设，培育发展医疗器械制造业。加快发展手机零部件产业，吸引终端整机制造企业落户。新材料新能源产业要依托易成新能、特耐、龙宇化工、华瑞新材、东磁新能源等企业，做强新型耐火材料、新型研磨材料及新型贮能材料，支持易成新能借力并购。以创新发展推动动能转换。大力支持企业科技创新，新建省市工程技术研究中心5—10家、市级重点实验室3家，支持安利达、茂盛粮机等企业与高校、科研机构共建研发平台。推动企业技术改造，出台奖补政策，制定工业新产品试制计划，组织产业共性关键技术攻关，提升产业素质。强力实施质量强市、标准化和品牌发展战略，力争再培育1—2家省长质量奖、5—7家市长质量奖企业，争创1—2个省质量强县（区）示范县（区），打造一批国家、省名牌产品和驰名商标。加强对企业家培训，注重培养创新精神和工匠精神。加快产业集聚区提质转型，推进尉氏、杞县、精细化工产业集聚区争创省级高新区，推进开封高新区争创国家级高新区，再打造一批专业化、高端化“园中园”。服务实体经济发展。继续推进“三去一降一补”，降低税负、用电、融资等十大领域的成本，取消、调整和规范一批行政事业性收费项目。引导支持企业通过直接融资降低杠杆率和财务成本，再实现9家企业上市融资。建立重点企业联席会议制度。继续开展“政策落实进千企”活动，常态化设置“首席企业服务员”，继续对“双50”企业、重点项目实行“一对一”服务。充分发挥企业投诉中心作用，帮助企业解决实际困难，提振企业发展信心。设立政府引导产业基金。今年至2020年，每年统筹整合财政金融资金5亿元作为产业引导基金，逐步形成总规模80亿至100亿的母基金群，撬动金融资本、社会资本300亿元以上。积极争取省各类产业基金支持，设立新能源汽车、先进制造业等产业子基金。

　　发展现代服务业。认真落实《推进服务业供给侧结构性改革专项行动方案》，发展新业态、培育新热点，力争服务业增加值增长12%以上。优先发展生产性服务业。借助自贸区建设机遇，加快打造金融“一区五平台”，实施“引金入汴”工程，力争引进20家金融、类金融机构。出台《发展服务外包产业行动计划（2017—2020）》，实施“586”工程，培育一批“专、精、特、新”中小型企业，积极创建省级服务外包产业示范园区。用好中国（郑州）跨境电子商务综合试验区政策，深入推进中国中部电子商务港、国家电子商务示范基地建设，大力发展电子商务。积极发展研发设计、信息技术服务、检验检测认证、商务咨询、工艺设计等生产性服务业。大力发展消费性服务业。实施“十大扩消费行动”，坚持满足需求和引导消费相结合，加快发展教育培训、健康养老、体育健身、绿色消费等新型消费业态，培育消费新市场。优化消费产品供给，改造升级“开封老字号”，培育壮大“开封新字号”，继续实施“双百”培育工程，推出一批消费新产品，培育新的消费增长点。着力发展特色服务业。树立全域旅游理念，坚持文商旅融合发展，促进七盛角、珠玑巷等服务业 “两区”完善业态、提升层次、聚集人气。全力推进恒大童世界项目实施，确保上半年开工建设。抓好万达商业广场、清明上河园三期、朱仙镇国家文化生态旅游示范区二期等文商旅项目建设。继续办好“两节一赛一论坛”。贯彻旅游行业标准，丰富文化旅游内涵，促进旅游接待量和旅游综合收入分别增长12%以上和13%以上。

　　发展现代农业。稳定提高粮食产能。再建成高标准粮田35万亩，确保粮食丰产丰收。持续抓好灌区续建配套与节水改造、中小河流治理、病险水闸加固等水利工程建设，完善引黄灌溉体系。提高农业机械化综合水平，持续推进气象防灾减灾和为农服务体系现代化建设。推动种养业供给侧结构性改革。按照“种养结构调优、加工能力调强、经营规模调大、产业链条调长”的要求，大力发展优质小麦、优质花生、优质草畜、优质果蔬、优质大蒜，适度压缩玉米种植面积，着力推进农业布局区域化、经营规模化、生产标准化、发展产业化，确保优质强筋小麦、优质油用食用花生、优质大蒜种植面积分别达到50万亩、100万亩、60万亩，以苜蓿为主的优质牧草达到1.5万亩以上，新增优质奶牛2000头、优质肉牛1万头。扭住 “全环节升级”、“全链条升值” 两个关键，继续完善9条农产品全产业链，推进一二三产融合发展。力争新增无公害农产品15个，绿色食品稳定在20个，使农业供给与市场需求更加契合。大力发展设施农业和休闲农业。制定出台设施农业发展实施方案，发展以优质果蔬为主的设施农业，力争设施农业达到9万亩。加快构建新型农业经营体系，加强农产品流通设施和市场建设。大力发展休闲农业与乡村旅游，首批培育10个休闲农业与乡村旅游示范项目，培育10个都市生态农业示范园区。

　　瞄准经济新业态。发展网络经济。融入国家大数据综合试验区战略，深入实施“互联网+”行动，加速制造业智能化服务化、种养业智慧化精准化、服务业品质化高端化。发展枢纽经济。用好新郑国际机场和郑州航空港经济综合实验区，加快打造铁路“两主两副”、公路四通八达的交通体系，启动6个大型物流园区规划建设，在7个产业集聚区谋划建设7个综合物流园区，聚集适宜在交通枢纽周边发展的特色产业。发展创意经济。着力发展与产业研发、文化旅游、服务消费、咨询策划相关的创意设计，推动优客工场入驻开封，加快960非遗文创园等创意项目建设，培育一批文化创意骨干企业和品牌。按照“政府引导、产业主导、企业主体、创新驱动、产城融合、项目支撑、生态共享”原则，启动特色小镇建设。发展生态经济。把绿色生态理念融入经济社会发展的方方面面，大力发展高效、低耗、环保的生态产业，推动环境保护与经济发展相生共赢。

　　（二）抓机遇、扩增量，拓展新的发展空间。紧盯自贸区开封片区、中原城市群核心区、开港经济带三大区域，汇聚拓展发展的新空间。

　　全力推进中国（河南）自由贸易试验区开封片区建设。准确定位，找准突破口。根据上报国家的中国（河南）自由贸易试验区总体方案，开封片区为19.94平方公里，重点发展服务外包、医疗旅游、创意设计、文化传媒、文化金融、艺术品交易、现代物流等服务业，提升装备制造、农副产品加工国际合作及贸易能力，构建国际文化贸易和人文旅游合作平台，打造服务贸易创新发展区和文创产业对外开放先行区，促进国际文化旅游融合发展。目前，开封片区属于非海关特殊监管区，要重点探索投资体制改革，完善事中事后监管，走出一条放管服“四个五”链条式服务新路子，带动全市现代服务业和高端制造业发展，为内陆地区开放发展创新机制积累可复制的经验。科学规划，高起点招商。按照功能定位，在片区内科学规划“一中心、六片区”，编制控制性详规，探索“多规合一”，对区域内现有业态进行调整，制定招商引资方案，面向海内外宣传，高起点招商，率先在CBD中心商务区和管委会行政服务中心落地一批项目，力争年底前形成不低于2000家金融、文化传媒、创意设计等现代服务业聚集的高地。加快开放，完善平台。加快设立国际贸易“单一窗口”、海关、检验检疫等机构，积极申建海关特殊监管区，发展口岸经济，建成国际艺术品交易中心等跨境贸易平台及保税仓。

　　全力推进郑汴深度融合发展。按照《中原城市群发展规划》，借助中原城市群核心区建设机遇，深入推进郑汴一体化“533”工程。加快“五条通道”建设。开工建设东京大道西延工程，力争一年内建成通车，打通郑汴产学研结合示范带通道。确保开港大道一期工程年底前建成通车，与郑州航空港迎宾大道贯通。启动郑汴沿黄大道一期工程（开封中牟交界—S213黄河大桥段），力争2018年年底前建成通车。做好G310南移项目前期工作，适时启动项目建设。着手编制开封轨道交通规划。推进“三大产业对接”。大力发展汽车及零部件产业，与郑州互补发展，加快打造具有较大影响力的汽车及零部件生产制造基地。加快郑汴旅游融合，打捆推介精品旅游项目，打造郑汴旅游城市圈。做好郑汴产学研结合示范带空间规划和产业规划，共同打造全省规模最大、最具活力的双创活力带。加强“三大区域协作”。统筹空间布局、产业发展、生态建设，推进自贸区郑州开封两个片区、“郑汴港”金三角和郑汴沿黄生态带的协同发展。同时，加快两市政务信息、文化、医疗卫生等各领域的信息资源共享。

　　全力推进开港经济带建设。去年，以开港经济带为基本区域，我市成功申报并获批首批国家产城融合示范区，要用好国家支持产城融合示范区发展政策，以汴西产业集聚区、朱仙镇文化旅游试验区、开港经济区“三个片区”为重点，支持示范区、祥符区、尉氏县在开港经济带总体规划下先行先试，推动开港经济带实质性建设。实施一批基础设施项目，确保开港大道二期启动，实现三个重点片区内基础设施“六通一平”。坚持轴线带动、分片区推进，根据产业规划定位，大力开展专项招商，启动项目布局，打造产业发展基础较好、城市服务功能完善、边界相对明晰的城市综合功能区。

　　（三）抓重点、促协调，推进新型城镇化。坚持以人的城镇化为核心，以提高质量为关键，持续推进“两核六组团”空间布局，加快新型城镇化建设步伐，力争常住人口城镇化率达到47%左右。

　　实施“两改两拆”，促进“双提双修”。继续实施“两改”。在“两改一建”的基础上，扎实推进24个已开工棚户区城中村改造项目建设，再启动实施27个项目，完成棚户区改造20916套（户），其中货币化安置率达到80%以上，原则上不再新开工建设保障性住房。强力推进“两拆”。制定三年滚动计划，实施以拆为主的综合整治措施，2017年清查、处理全市建成区违法建设的35%，到2019年基本清查、处理完毕，违法用地发现一起查处一起，坚决遏制新增违法建设行为。促进“双提”。加快基础设施提质、市容环境提升，全面实施百城建设提质工程。以此为契机，祥符区、尉氏县、杞县、通许县创新融资思路，实施一批道路、供排水、燃气、供热、园林绿化等基础设施提质项目，解决县城基础设施落后、规划无序、环境面貌不佳等问题。市区以“6+6”示范街全面整治提升为带动，解决城市重建轻管的现象，稳妥推进城市执法体制改革，制定城市管理权责清单，基本完成机构设置，逐步建立“1+8”一体化城市执法管理平台，全面实施县城数字化管理，制定数字城管向智慧城管转变方案，加快建设智慧化城市管理平台。开展城市“双修”。出台《古城保护与更新改造管理办法》，推动城市修补和生态修复，老城建设重点实施古城修复，加快城墙贯通、水系贯通，推进双龙巷、徐府街、大相国寺等重点片区改造，争创国家城市“双修”试点。

　　继续完善八网基础设施。完善路网。实施“四路四互通”工程，完善开杞路、开尉路，启动开柳路、开通路改造，开工连霍高速十三大街、开柳路互通口建设，做好郑民高速开通路、机场东路互通口前期工作，逐步完善“两核六组团”之间的快速路网；加快郑开城际铁路延长线项目建设；建成夷山大街（土城段）、迎宾路（省府西街—西门大街）等在建道路，开工万膳街（西司桥—西门大街）、黄河大街北延（复兴大道—复兴北路）、宏达大道（新曹路—新宋路）、夷山大街北延（东京大道—复兴大道）、西关北街北延（金耀路—东京大道）、机场东路一期（310国道—华夏大道）等6条市政道路建设，改造提升内顺城路（迎宾门—大南门）、包公湖南路（迎宾路—包公湖泵站）、陵园路（惠济河桥—烈士陵园南门东现状路）等3条市政道路，加快打通“断头路”。完善水网。加强饮用水源地保护和整治，加快黑池、柳池清淤，推进引水渠明渠改暗渠；实施南水北调入汴工程，力争年底实质性开工，合理布局供水管网建设，让人民喝到优质水。建成汴京路、西环路等雨水泵站，提升城市防汛排涝能力。启动“一渠六河”综合整治，完成涧水河项目主体工程，力争三年内实现新老城区十湖连通。完善热网。解决东区、北区和老城区供暖问题，实施“煤改电”工程，市区新增供暖面积不少于400万平方米，建成区居民集中供热率提高到60%以上，稳步扩大县城供暖面积。抓紧布局市区清洁煤场生产销售网点，确保清洁煤源供应。完善气网。实施“煤改气”工程，确保城区燃气气化率达到95%以上，县城燃气气化率达到60%以上。完善电网。建成44个10千伏打捆项目，新建续建12个110千伏及以上输变电工程，全面完成新一轮农村电网提升任务。完善绿网。持续开展高速公路绿化廊道建设，实施汴西森林公园改造提升、植物园等项目，积极开展园林县城、园林城镇创建。完善云网。实施“宽带开封”战略，确保城市家庭宽带接入能力达到50兆比特每秒以上。完善提升大数据中心，建成应急指挥中心，进一步整合政务云、旅游云、文化云、服务云、交通云等云端资源。完善管网。统筹地下、地上，做好地下综合管廊规划，在城乡一体化示范区开工建设10公里地下综合管廊。

　　促进房地产市场平稳健康发展。继续加大房地产去库存力度，确保房地产去库存周期不超过12个月。再盘活10个以上的商品房烂尾项目。坚持“房子是用来住的、不是用来炒的”定位，加强房地产市场顶层设计，统筹安排土地供应、住房建设总量和开发时序，加强市场调控。用好郑徐高铁开通运营、郑开城际铁路增加班次机遇，活跃房地产市场。试点先行，建设新型装配式钢结构住宅。基本解决竣工验收备案一年以上未分配公租房问题，出台《存量房交易合同网签和交易资金管理办法》，加快构建市场配置与政府保障、自助购买与市场租赁相结合的住房体系。

　　积极推进农业转移人口市民化。加大城乡统筹力度，健全社会保障制度，深化户籍制度改革和居住证制度改革，建立农业转移人口市民化激励机制，用好城镇建设用地增加与吸纳农业转移人口落户数量挂钩政策，维护好进城农民土地承包权、宅基地使用权、集体收益分配权，争取新增农业转移人口7.5万人以上。

　　（四）蓄势能、强支撑，增强经济发展的动力与活力。破解产业结构不优、新旧动能转换乏力、体制机制不活等问题，关键要激发内生动力，提升竞争新优势。

　　强化创新驱动。培育创新主体。继续实施“科技小巨人”、高新技术企业培育计划，新培育科技型中小企业100家，力争达到500家以上，新培育高新技术企业15家，力争达到50家以上。打造创新平台。新建一批众创空间、科技企业孵化器。落实好与清华大学、天津大学等高校及科研院所签订的战略协议，培育新型研发机构和院士工作站。加快建设大学科技园。充分利用河南大学的优势，做好河南大学的技术转化承接。理顺创新机制。落实好《实施创新驱动 打造科技创新高地活力区行动方案》，加快科技项目管理改革，推进科技金融结合，打破影响创新创业的体制机制障碍。培养创新人才。大力实施创新型科技人才工程，制定具有开封特色吸引力的人才政策，引进科技创新领军人才10人以上，培育市级科技创新团队10个左右。

　　强化改革推动。打好国企改革攻坚战。稳妥推进国有工业企业、国有非工业企业改革，推进投融资企业现代化运营管理，深化企业内部三项制度改革，按时间节点完成驻汴央企、省企 “三供一业”分离移交工作，加强国有资产监管，防止国有资产流失。“放管服”改革走在前列。先行在自贸区内大胆探索行政审批制度改革，继而面向全市复制，开展“减证便民”专项行动，率先在全省建立“互联网+政务服务”体系。强化事中事后监管，实现“双随机、一公开”监管方式全覆盖。推进投融资体制改革。用好财政部和省PPP基金支持政策，积极探索“基金+PPP”模式。用好“浦银汴投城市运营发展基金”、“开封市城市发展基金”，积极争取邮银豫资“一带一路”（河南）发展基金支持，稳步扩大基金、信托融资规模。深化医药卫生体制改革。全面实施分级诊疗制度，开展健康签约服务，构建科学合理的双向转诊机制。启动城市公立医院改革，加强医联体建设。抓好基本药物制度落实，继续实施药品零差率销售。推进基本医保支付制度改革，建立稳定可持续的筹资和补偿比例调整机制。同时，落实好财税、农村、科技、文化、电力、价格、社保、司法、事业单位等领域的各项改革。

　　强化项目拉动。实施重点项目“5412”工程，围绕“十大工程、八网基础设施、重点产业、文商旅综合体、民生实事”五大类，实施400个左右的重点项目，力争全年完成项目投资1200亿元左右。持续实施重大项目推进“六项机制”，坚持重大项目联席会议制度，跟踪协调解决项目建设中存在的困难和问题，确保重点项目按时推进、顺利竣工。

　　强化开放带动。进一步转变招商理念、完善招商链条，以自贸区开封片区为引领，加大招商引资力度，力争新引进省外资金项目240个，实际到位省外资金510亿元，确保近两年来招商引资项目落地率达到60%。继续开展县区招商“532”工程，力争引进35个投资5亿元以上的重大项目。加强对外经济技术合作，进一步扩大外贸出口规模。继续实施抓大扶新措施，抓好外经企业培育，扩大“走出去”企业数量和规模。

　　强化风险防控。狠抓安全生产责任落实，严格隐患排查整改，启动安全生产综合服务和技术支撑基地建设，有效防范和坚决遏制重特大事故发生，实现事故总量、死亡人数“双下降”。加强食品药品监管能力建设，积极推进“四个一工程”，持续开展食品药品综合整治行动，加强农副产品和进口食品监管，争创国家食品安全示范城市。加强防灾减灾体系建设，建设突发公共事件预警信息发布系统，提高防灾减灾和应急救援能力。依法依规处置非法集资案件，最大限度维护群众利益。完善信访工作机制，深化矛盾纠纷调处化解，做好重大项目社会稳定风险评估，强化社会诚信体系建设，及时预防化解各类群体性事件。

　　（五）惠民生、促和谐，共享“四个开封”建设新成果。坚持以人民为中心的发展思想，以人民群众关心关注的热点难点问题为重点，办好民生实事。

　　在“实力开封”建设中提升人民群众的获得感。加大民生投入，继续实施十项民生工程50件为民实事，确保一般公共预算支出70%以上用于民生、保障民生。深入推进脱贫攻坚战。按照《“十三五”脱贫攻坚规划》《脱贫攻坚四年滚动脱贫计划》，落实好脱贫攻坚系列政策措施和“十不十有”标准，研究非贫困县贫困村、非贫困村贫困户脱贫工作，高质量完成66个贫困村，3万农村贫困人口脱贫。着力扩大就业。深入实施全民技能振兴工程，支持大学生和农民工返乡创业，申报建设省级高技能人才培训示范基地、省级创业培训示范基地、开封市公共实训基地，确保城镇新增就业5.5万人以上，城镇登记失业率控制在4.5%以内。持续增加农民收入。培育新型农业经营主体，健全农业社会化服务体系，加快发展农村电商、乡村旅游等新业态新模式，强化技能培训，完成农民转移就业2.6万人，落实财政补贴，推广农业保险，帮助农民避灾减损。改善群众出行条件。有效缓解交通拥堵，对10个交叉路口进行交通渠化改造，新建公共停车场泊位4000个。优先发展公共交通，启动公交都市创建工作，加快充电桩布局，统筹公交场站建设，确保610辆新能源纯电动公交车高效运营，再优化调整15条公交线路，探索定制公交、私家车错峰出行等措施。深化出租汽车行业改革，明确网约车合法地位，鼓励私人小客车合乘出行。新改建农村公路640公里、桥梁940延米。着力健全公共服务。推进全民健身和全民健康深度融合，提高竞技体育水平。加快建设环城墙健身带，建成200处农民体育健身工程，积极争创全国武术之乡。编制市场建设规划，完成东京大市场搬迁，建成劳动路市场，开工建设香港昌升等一批专业市场。

　　在“文化开封”建设中提升人民群众的自信感。继续实施“文化+”战略。打造文化高地示范区。大力弘扬焦裕禄精神，学习王振立等先进典型事迹，提升和完善一批思想道德教育基地。强化文化事业文化产业人才队伍建设，加强宋文化研究，推出一批新的文化研究成果，积极参与中华文化“走出去”工程。开展开封文化奖章评选活动。加强互联网管理，持续打造清朗网络空间。提升市民文化素养、文明素质，认真落实“十大开封规范”，做好国家卫生城市复核工作，力争成功创建全国文明城市。提升公共文化服务水平。加大政府购买公共文化服务力度，推动公共文化服务设施免费开放。筹建公共图书馆、文化馆等公共设施，开放博物馆、规划馆、美术馆新馆。完善县乡村三级文化网络，加快乡镇综合文化站、农家书屋、农村文化广场等公共文化服务设施建设。大力倡导全民阅读，丰富群众精神生活。实施一批文化项目。认真落实《宋都古城风貌保护规划》，牢固树立“新宋风”理念，做精做足宋文化品牌，加强传统街区保护、古城文脉传承，大力推进西湖东岸仿古建筑群、市文化交流中心、文化艺术品综合交易平台等文化项目建设，推动文化事业和文化产业共同发展。

　　在“美丽开封”建设中提升人民群众的宜居感。打好环境治理攻坚战。持续打好大气污染防治攻坚战，力争PM2.5浓度均值不高于63微克/立方米，PM10浓度均值低于104微克/立方米，大气优良天数不低于213天。打好水污染防治攻坚战，系统推进水污染防治、水生态保护和水资源管理，推行“河长制”，加强对惠济河、贾鲁河、涡河等流域综合整治，完成东干渠、化肥河等6条黑臭水体治理，全面消除建成区黑臭水体；实施雨污分流，推行城市排水许可制度，加快海绵城市示范区建设。认真落实国务院“土十条”，加强土壤污染治理与修复，推进重金属污染物总量减排，严格危险废物环境监管，还人民群众一个“天蓝地绿水净”的生活环境。持续加强生态建设。加强农田防护林等工程建设，启动国家森林城市创建工作。推进湿地生态保护与开发利用协调发展，启动沿黄湿地公园建设。加大节能减排力度。推进大宗固废综合利用，实施餐厨废弃物资源化利用和无害化处理项目，再建成一批生活垃圾中转站。着力改善农村人居环境。开展农村环境卫生整治和农村生活垃圾治理专项行动，梯次推进农村生活污水治理，建立健全农村环境卫生治理长效机制。深入开展美丽乡村创建，按照“科学规划布局美、村容整洁环境美、创业增收生活美、乡风文明素质美”的要求，再建15个美丽宜居乡镇、50个美丽宜居村庄。

　　在“幸福开封”建设中提升人民群众的安全感。完善社会保障体系。继续实施全民参保登记计划，加快推动社会保障由制度全覆盖转向法定人员全覆盖。全面完成机关事业单位养老保险制度改革和城乡居民基本医疗保险整合，提高城乡居民基本养老保险金标准。统一城乡居民医保制度，财政补贴标准提高到每人每年450元。加快五福爱心护理院、民生养老院、示范区福利中心建设，建成开封市康复医院和10家社区老年人日间照料中心。健全社会救助、社会福利和养老服务体系，加强农村“三留守”人员关爱服务和困境儿童保障。努力办人民满意的教育。注重教育内涵式发展，加大对校长和教师专业化培训，加快义务教育发展基本均衡县（区）创建工作，推动教育向高位优质均衡发展。启动河南大学附属中小学、幼儿园建设，再新建中小学校10所、改扩建中小学校8所。大力发展普惠性幼儿园，加快普及高中阶段教育。支持河南大学、黄河水利职业技术学院等高校发展，办好开封大学、开封文化艺术职业学院。推进职业教育园区建设，加快职业教育发展。完善助学资助体系，让更多贫困家庭孩子有机会接受更好教育。全面提高医疗健康保障水平。优化城市医疗卫生资源结构，推进市中心医院新院区、市中医院全科医师培训基地等10个医疗卫生项目建设。提升公共卫生保障水平，健全疾控体系，强化卫生应急和医疗急救覆盖能力。大力推进健康服务业，完善护理服务体系，积极推广中医药适用技术，促进医养结合发展。开展“智慧医疗”建设，持续提升医疗服务质量，方便群众看病就医。强化社会治安防控体系建设。开工建设市消防应急救援训练基地、警训和特警支队干警训练基地等项目，建成350兆警用数字无线电通信系统，安装1000路社会防控摄像头，着力提高动态化、信息化条件下驾驭社会治安局势的能力。

　　继续加强国防动员、国防教育和国防后备力量建设，支持开封驻军、武警部队、预备役部队改革，切实抓好民兵和人民防空建设，深入开展双拥共建，巩固军政军民团结，推动军民深度融合发展。支持工会、共青团、妇联等人民团体充分发挥桥梁纽带作用，注重发挥工商联等社会团体作用。大力推进民族团结进步事业，依法管理民族宗教事务。加强统计、督查工作。继续做好外事、侨务、港澳、对台、史志、地震、档案、社科研究、援疆等工作。

　　四、全面加强政府自身建设

　　“大道至简，落实为要”，再好的谋划也不如坚决执行、马上行动。全面加强政府自身建设，坚决克服“能力不足不会为、动力不足不想为、担当不足不敢为”问题，坚决消除“思考问题肤浅、工作作风浮躁、总结汇报浮夸”现象，不断提升工作水平、提高工作效率，更好地服务开封人民。

　　（一）牢固树立“四个意识”。始终与以习近平同志为核心的党中央保持高度一致，切实增强“四个意识”特别是核心意识、看齐意识，政治上坚决维护核心，组织上自觉服从核心，行动上坚定紧跟核心。严守政治纪律，恪守政治规矩，保持政治定力，自觉坚持党的基本路线，自觉贯彻党的理论和方针政策，自觉落实党中央、国务院和省委、省政府及市委的决策部署，凡是党中央提倡的坚决响应，凡是党中央决定的坚决执行，凡是党中央禁止的坚决不做，坚决维护党中央权威。

　　（二）坚持解放思想。开封依靠解放思想才有了今天的发展，只有进一步解放思想才会有更加美好的未来。打破思想禁锢，开阔视野眼界，都要不断学习，要结合工作加强学习，避免少知而迷、无知而乱，熟悉新理念、新技术、新业态、新商业模式，提高政府系统干部的专业思维、专业素养、专业方法，学习借鉴先进地区发展经验，让“远学杭州、近学徐州、融入郑州”变成真正的行动和自觉，而不是停留在口头上。

　　（三）严格依法行政。把法治思维和法治方式贯穿于政府工作全过程，严格按照法定权限和程序行使权力、履行职责。全面推行政府法律顾问制度，加强智库建设，健全依法决策机制，严格履行公众参与、专家论证、风险评估、合法性审查和集体讨论决定等重大行政决策法定程序。切实加强政府立法，为城乡建设管理、环境和历史文化保护提供更多法治保障。全面推行政务公开，加强政府信用建设，言必信、行必果。依法接受人大法律监督，自觉接受政协民主监督，认真办理人大代表建议和政协提案，广泛听取民主党派、工商联、无党派人士、人民团体意见，自觉接受社会监督，让权力在阳光下运行。

　　（四）转变工作作风。“天上不会掉馅饼”，推动开封发展要想干事、会干事、敢干事。继续秉持“干净干事”的工作理念，“有激情、有自信、有定力”的工作状态，“抓优势、抓短板、抓重点”的工作方法，坚持沉下去解决问题、走出去招商引资、下力气化解风险、静下心研究问题。完善抓落实工作机制，把抓落实、求实效作为衡量工作成效的重要标准，对工作落实不力、效能低下、敷衍塞责、弄虚作假等行为，严肃追责问责。要建立有效机制，激励广大公职人员尽职尽责，敢于担当，苦干实干加油干，不辜负全市人民的信任与期待。

　　（五）坚持清正廉洁。严格落实各级政府党组主体责任，认真执行《新形势下党内政治生活的若干准则》和《中国共产党党内监督条例》，强化正风肃纪，严格责任追究。强化行政监察和审计监督，重点加强公共资源交易、政府投融资等监管。深入落实中央八项规定精神、省委20条意见和市委16条意见，严格财经纪律，严控“三公”消费，坚决防止“四风”反弹回潮。

　　各位代表！

　　梦想承载希望，实干开创未来。虽然今年各项工作任务繁重而艰巨，但有全市广大干部群众希望加快发展的思想共识，有我市面临的重大机遇和日益凸显的发展优势，有我们对自身问题差距的清醒认识，有焦裕禄精神的激励与鼓舞，我们对完成今年目标任务充满信心！

　　各位代表！

　　让我们更加紧密地团结在以习近平同志为核心的党中央周围，在中央、省委和市委的坚强领导下，为加快建设“四个开封”、决胜全面小康作出新的更大贡献，以优异成绩迎接党的十九大胜利召开！
